# Membranous Dysmenorrhœa

**Published:** 1878-07

**Authors:** 


Membranous Dysmenorrhœa.—Dr. Ranking reports a case of membranous dysmenorrhœa that was much benefited by the application of Chapman's spinal hot-bag to the lumbar region for two hours three times a day, and ten drops of Donovan's solution taken internally three times daily for three months. The general effects produced were diminished formation and thickening of the membrane, it becoming less leathery and more distinctly cellular; increased facility of separation of the membrane, and more rapid and complete expulsion; shortening of the menstrual period from ten to four days; more rapid recovery, in consequence of the diminution of pain and uterine action; benefit indirectly accruing from diminished bruising of the cervical canal; smaller quantity of morphia required; no iodism or mercurialism.—*British Medical Journal.*

